# Prevention, screening and treatment of colorectal cancer: a global and regional generalized cost effectiveness analysis

**DOI:** 10.1186/1478-7547-8-2

**Published:** 2010-03-17

**Authors:** Gary M Ginsberg, Stephen S Lim, Jeremy A Lauer, Benjamin P Johns, Cecilia R Sepulveda

**Affiliations:** 1 Costs, Effectiveness, Expenditure and Priority Setting, World Health Organization, Geneva, Switzerland; 2 Chronic Diseases Prevention and Management, World Health Organization, Geneva, Switzerland

## Abstract

**Background:**

Regional generalized cost-effectiveness estimates of prevention, screening and treatment interventions for colorectal cancer are presented.

**Methods:**

Standardised WHO-CHOICE methodology was used. A colorectal cancer model was employed to provide estimates of screening and treatment effectiveness. Intervention effectiveness was determined via a population state-transition model (PopMod) that simulates the evolution of a sub-regional population accounting for births, deaths and disease epidemiology. Economic costs of procedures and treatment were estimated, including programme overhead and training costs.

**Results:**

In regions characterised by high income, low mortality and high existing treatment coverage, the addition of screening to the current high treatment levels is very cost-effective, although no particular intervention stands out in cost-effectiveness terms relative to the others.

In regions characterised by low income, low mortality with existing treatment coverage around 50%, expanding treatment with or without screening is cost-effective or very cost-effective. Abandoning treatment in favour of screening (no treatment scenario) would not be cost effective.

In regions characterised by low income, high mortality and low treatment levels, the most cost-effective intervention is expanding treatment.

**Conclusions:**

From a cost-effectiveness standpoint, screening programmes should be expanded in developed regions and treatment programmes should be established for colorectal cancer in regions with low treatment coverage.

## Background

In 2000, colorectal cancer accounted for approximately 579,000 deaths (equivalent to 1% of all deaths and 8% of deaths due to malignant neoplasms) worldwide. In burden-of-disease terms, colorectal cancer accounts for 0.38% of all DALYs and 7.2% of DALYs due to malignant neoplasms [1]. Geographical disparities in the burden of colorectal cancer are pronounced. For example, colorectal cancer incidence rates are 5-10 times higher in the most developed regions of the world than in developing regions (personal communication, K.Shibuya, World Health Organization).

Cost effectiveness analyses of the many interventions (primary prevention, screening or treatment) for reducing the burden of colorectal cancer have usually been restricted to developed country settings and with often considerable variation in the analytical methods used. This limits the value of the existing literature to inform colorectal cancer control policies in low to middle-income country settings. Assessment of costs and effects of different strategies can help guide decisions on the allocation of resources across interventions, as well as between interventions for colorectal cancer and interventions for other conditions or risk factors.

This research presents estimates on the costs and effects of various combinations of available intervention strategies for colorectal cancer across regions using standardised methods, data sources and tools [2-10] that have been developed by the WHO-CHOICE (*CHO*osing *I*nterventions that are *C*ost *E*ffective) program. The results should help answer policy questions such as whether and what type of screening programmes should be added in populations with a high level of access to treatment, or, in developing countries, whether to put scarce resources into screening or into expanding levels of treatment coverage. In addition, the study will facilitate the prioritisation process by allowing comparisons to be made, using similar methodologies, with interventions (whether, primary prevention, screening or treatment) for cardiovascular [11] and other diseases [12].

## Methods

### WHO-CHOICE framework

WHO-CHOICE comprises sectoral, population-level cost-effectiveness analyses based on a generalized cost-effectiveness analysis framework [6]. Generalized cost-effectiveness analysis is characterized by the assessment of costs and effects against a reference scenario defined as the absence of all current interventions against the disease or risk factor (the "null scenario"). This approach facilitates [13] the comparison of cost-effectiveness findings across competing interventions [14].

Costs and effects of key interventions for colorectal cancer were modeled at the population level in 14 WHO regions [15].

Basically there are two stages to the calculations:-

i) We first constructed a model that predicted intervention-specific decreases in incidence and case fatality rates.

ii) The data from the first model was then combined with regional specific demographic and cost data and run over a time period of one hundred years in order to predict regional intervention specific outcomes in terms of costs and DALYs saved.

### Choice of interventions

The interventions analyzed are listed in Table 1 represent protocols that are either recommended [16] or used in some countries [17] or combinations there of. The reference strategy in keeping with the methodology of generalized cost-effectiveness analysis is the null consisting of no intervention or treatment.

**Table 1:** Estimated Effects of Interventions (based on model of AMRA region) and assumed Compliance data that were inputted into POPMOD model.

Intervention	Description	Decrease in Incidence	Decrease in Case-Fatality Rate	Compliance
FOB1	Annual Fecal Occult Blood Tests^a^	35.0%	0%	56.8%

FOB2	Biannual Fecal Occult Blood Tests^a^	21.9%	0%	61.8%

SIG5	Sigmoidoscopy every 5 years[1]^a^	38.9%	0%	45.0%

COL10	Colonoscopy every 10 years^a^	52.6%	0%	45.0%

FOB1SIG5	Annual FOBT, SIG every 5 years^a^	51.5%	0%	45.0%

FOB50	FOBT at age 50^a^	2.6%	0%	71.8%

SIG50	Sigmoidoscopy at age 50^a^	11.8%	0%	55.0%

COL50	Colonoscopy at age 50^a^	25.9%	0%	55.0%

FOBSIG50	FOBT & SIG at age 50^a^	13.2%	0%	55.0%

RX	Medical Treatment of cancers^b^	0%	91.9%	100%

FOB1RX	Combination of FOB1 & RX	35.0%	17.9%^c^	56.8%

FOB2RX	Combination of FOB2 & RX	21.9%	12.9%^c^	61.8%

SIG5RX	Combination of SIG5 & RX	38.9%	3.4%^c^	45.0%

COL10RX	Combination of COL10 & RX	52.6%	3.9%^c^	45.0%

FOB1SIG5RX	Combination of FOB1SIG5 & RX	51.5%	18.3%^c^	45.0%

FOB50RX	Combination of FOB50 & RX	2.6%	0.5%^c^	71.8%

SIG50RX	Combination of SIG50& RX	11.8%	0.3%^c^	55.0%

COL50RX	Combination of COL50& RX	25.9%	0.4%^c^	55.0%

FOBSIG50RX	Combination of FOBSIG50 & RX	13.2%	0.5%^c^	55.0%

FVCAMP	Fruit & Vegetables campaign	d)	0%	---

FVCAMPRX	Combination of FVCAMP & RX	d)	0%^c^	---

DRE1	Digital Rectal Exam annually^a^	17.6%	0%	50%

DRE1RX	Combination of DRE1 & RX	17.6%	1.8%^c^	50%

Notes:

Subscripts 1,2,5,10 (eg: SIG5] in the intervention column denote the frequency of screening in years.

Subscript 50, denotes a one off intervention at age 50.

RX denotes the availabily of treatments for cancers in addition to the intervention program.

Efficacy varied slightly between regions due to demographic differences.

Efficacy considered on an age-sex specific basis.

a) Denotes colonoscopy performed on all positive tests, with subsequent removal of lesions or polyps if discovered.

b) Including surgical, radiotherapy and chemotherapy.

c) In excess of decrease in CFR caused by treatment.

d) Varies by region

These can be grouped into the following categories:-

#### Repeated Screening (followed by removal of polyps or potentially cancerous lesions)

i) Five interventions represent longitudinal screening programs based on current consensus recommendations [16]. These interventions (Annual and Biannual FOBT, Sigmoidoscopy every 5 years, Colonoscopy every 10 years and Annual FOBT with Sigmoidoscopy every 5 years) are analysed first in a scenario where no treatment (radiotherapy, surgery or chemotherapy) for cancers is available. Individuals screened positives are assumed to have follow-up colonoscopy with the removal of any detected polyps or lesions.

#### One-off Screening (with polyp and lesion removal)

ii) Four additional interventions (FOBT, Sigmoidoscopy, Colonoscopy and Annual FOBT with Sigmoidoscopy combined) represent a one-off screening program (with polyp and lesion removal) for persons aged 50 years, akin to the sigmoidoscopy program recently introduced in France.

#### Treatment

iii) Treatment interventions include combinations of surgery, radiotherapy and chemotherapy, consistent with current practice in developed countries.

### Repeated Screening (with polyp and lesion removal) and treatment

iv) A combination intervention consisting of each of the five repeated screening programs in a scenario where treatment is available.

#### One-Off Screening (with polyp and lesion removal) and treatment

v) A combination intervention consisting of each of the four one-off screening programs at age 50 in a scenario where treatment is available.

#### Prevention

vi) Increasing fruit and vegetable consumption by means of mass media campaigns. The cost-effectiveness of this intervention is likely to be underestimated in this analysis as the likely benefits of decreases in other diseases, such as cardiovascular disease and strokes, was beyond this analysis's scope [17].

#### Other interventions of uncertain efficacy

vii) The final two interventions are annual Digital Rectal Exams (DRE) with and without medical treatment. These were included because of its "low-technological" approach for possible use in developing countries, despite the fact that evidence for this intervention vis-à-vis colorectal cancer is based on non-significant results from a lone case-control [18]. Despite not being recommended in most developed countries, results have been presented for comparative completeness. While we included benefits of DRE of reducing colorectal cancer, we did not include any possible benefits resulting from reducing prostate cancer.

#### Interventions not included

Double contrast barium enema was not analyzed due to the lack of evidence of reductions in incidence or mortality [19-22]. Furthermore, barium screening has low sensitivity for diagnosing symptomatic patients [19] and polyps [23] and hence limited applicability to population screening. Finally, compliance is likely to be low due to the perceived unpleasant nature of the test [22].

Despite the availability of data on consumption and price elasticity [24,25], price subsidies to increase fruit and vegetable consumption were not analyzed due to theoretical difficulties in calculating intervention costs in economic terms (since subsidies are transfer payments from the government to consumers). A further complication is a possible increase in red meat consumption, itself a potential risk factor for colorectal cancer [26,27], due to income effects.

Other preventive interventions like mass media campaigns to increase physical activity [28,29] and reduce body mass index were excluded because of insufficient data on the large-scale efficacy of such campaigns. The effects of changing transport modes (e.g. increasing rail and bike travel) and of urban planning (eg. decreasing "sprawl") on physical activity were also excluded due to lack of time-series data [30,31].

Reducing tobacco use was not considered because available evidence is insufficient to show a causal link with colorectal cancer [32]. Lack of data on efficacy was the primary reason for excluding palliative care for late-stage cancers.

Aspirin [33] or Folic Acid [34] were not considered as potential interventions because evidence for their efficacy is only based on case-control and cohort studies. This level of evidence does not meet the WHO-CHOICE requirement of evidence from randomized controlled trials in order to evaluate pharmacological interventions.

### Estimates of efficacy of interventions (Table 1]

To date, there have only been four randomized trials on Fecal Occult Blood Tests [35] (FOBT), the longest trial based on 18 years of follow up [36] reported decreases in incidence of colorectal cancer of 20% and 17% for annual and biennial screening respectively. Since these randomized trials reported results of guaiac FOBT as opposed to immunological tests, all the results in this paper relate to guaiac FOBT testing. Results from current randomized sigmoidoscopy trials (a once-per-lifetime study performed in the UK and a penta-annual USA study that included additional annual FOBT testing), are not yet published. To date, there have been no randomized trials of colonoscopy.

Evidence is not available from randomized trials of the efficacy of various screening interventions (except for FOBT). Therefore researchers often rely on modeling techniques in order to estimate the effects of screening for colorectal cancer. As a result of variations in quality, specification and parameter values, model results vary considerably (as detailed in the opening paragraph of the discussion).

Since no single model can be regarded as a "gold-standard", we constructed our ownmodel using a spreadsheet to estimate the effects of various screening interventions aimed at the general population aged 50 to 80 years old. The model allowed for examining the effects of varying the frequency of screening and age at time of screening. This model was based on demographic data from the WHO AmrA region (i.e. Canada, Cuba and the USA) and colorectal cancer incidence rates from the SEER registry in the USA for the period 1995-2000 [37]. Age-specific polyp incidence was estimated from prevalence data based on the weighted average polyp prevalence from studies on populations in the USA [38-46].

Age-specific rates of cancers originating in adenomateous polyps were calculated under the consensus-based assumption that 70% of cancers originated in adenomateous polyps [47,48] and that the average waiting time for development of cancer was ten years [22,47-50] (assumed normally distributed with a standard deviation of four years). The incidence of polyps was matched with future incidence of cancers originating from polyps in order to calculate the conversion rates from polyps to cancers, taking into account intervening mortality. Thus a proportion of polyps at each stage were assumed to be potentially carcinogenic and placed in a waiting state from which they were allowed to become malignant at a constant rate. Cancers were assumed to wait for two years in stage A and for one year in each of the three subsequent stages, if left untreated [47,51,52].

Using stage-specific fatality rates, the expected number of cancer cases and cancer fatality were estimated under a baseline scenario of no screening. Data on sensitivity and specificity of screening [47] was used to estimate the number of persons undergoing follow-up colonoscopy (assuming 100% compliance after a positive test) and the number undergoing polypectomy during the colonoscopy. For each intervention, based on the sensitivity, specificity and frequency of screening, the model estimated the number of polyps that would progress to cancers.

Despite their being some misgivings [53], our model was based on the mainstream accepted wisdom [54] that screening enables detection and removal of potentially cancerous polyps, thereby reducing the incidence of colorectal cancer even when cancer treatment was not available.

When medical treatment is available, screening enables detection of cancers at an earlier less-severe stage, thus reducing case-fatality rates (CFR). It was assumed that persons screened positive in areas which lack availability of treatment will only benefit via reduction in incidence (via polyp removal) and not via decreases in case-fatality rate due to the lack of treatment. We assumed that there would not be a change to more frequent protocols in persons who had a polyp removed.

These modeled intervention-specific estimates of CFR reductions, together with estimates of incidence reductions (Table 1) form the main inputs into a population based model described later on in this article.

The effectiveness of the fruit and vegetable campaign was calculated from the results of the campaign in Victoria, Australia [55], which achieved an increased intake of around 12.4% by weight in fruit and vegetable consumption. Assuming each 80 mg increase in average regional daily consumption results in a 1% decrease [95%CI, -2%, +3%) in colorectal cancer risk [24], this translates into risk reductions ranging from 0.34% in South America to 0.78% in Western Europe.

### Validation of model

For a specific validation of the model, the estimated decrease in incidence due to annual FOBT screening was found to be almost equal to benchmark data from 18-year follow up of the randomized controlled trial after adjustment for the period during the trial when screening was temporarily halted, as well as adjustment for compliance [36].

For general validity, across the various interventions, the estimated decreases in incidence and fatality over and above that due to treatment (Table 1) fell within the 25th and 75^th ^percentile range of the many modeled studies [47,49,56-73].

### Compliancy

The effects of each intervention were modified by their specific adherence or compliancy. The estimated magnitude of compliancy that was calibrated into the model was based on reported compliancy and assumptions as follows:-

Information on compliance with FOBT screening protocols were obtained from a demonstration project for annual screening [74] (i.e. 56.8%); biannual screening was assumed to result in 5% higher compliance. Compliance with screening by colonoscopy every 10 years, as well as annual FOTB combined with sigmoidoscopy every 5 years, was assumed to be the same as that found for a pre-intervention pilot study for sigmoidoscopy [75] (i.e. 45%), the greater invasiveness and more intensive preparations required for colonoscopy were assumed to be balanced by the longer interval required between screenings. Estimates of compliance for one-off screening at age 50 years was assumed to be 10% higher than that for repeated screening starting at age 50 and finishing at age 80 (Table 1). Due to the difficulties of estimating compliancy over a 30 year period, involving between 4 and 30 screening visits, all estimates of compliancy used in the model should be viewed as rough approximations. Intervention effectiveness was adjusted for the compliance assuming a target coverage rate of 100% for all regions.

### Definition of the null scenario

There is little direct evidence regarding the natural history of colorectal cancer in the absence of treatment. One small study in the USA found a 4.2% ten-year survival rate in persons who refused treatment (n = 24), for unstated reasons [76].

Our estimates of regional cancer incidence, mortality and remission rates were based on aggregated country data from the WHO. In countries where mortality data was incompletely reported, the WHO proxied estimates of cancer mortality by estimating survival data based on a function of the level of economic development of the specific countries [77,78].

AfrE and AmrA have low and high remission rates as a result of their treatment coverage rates (in the 30-69 age group) being respectively low [6.7%) and high [95%-100%). Linear extrapolations were made to this data in order to estimate age-and-sex-specific remission (and hence ten-year fatality) rates in the absence of treatment (ie: 0% treated).

Ten-year remission and fatality rates were converted to annual hazards according to the following formulas [79]:

Similarly, based on data from the AmrA region, where treatment coverage ranged from 90%-100%, linear extrapolations were made to estimate age-and-sex-specific ten-year fatality and remission rates assuming complete treatment of all colorectal cancers. The resutling estimates of overall remission and fatality rates were used for the various analysed treatment scenarios.

In 2000, the AmrA region of WHO was the only region globally where any significant level of population screening for colorectal cancer was being carried out (personal communication, Wendy Atkin, UK Colorectal Cancer Unit, St Marks Hospital, Middlesex). Based on modeled estimates of the effectiveness of screening, the observed incidence of colorectal cancer in AmrA was adjusted to reflect the higher incidence that would have occurred if a small percentage of the population had not been screened [80].

### Population Model (PopMod) for colorectal cancer

**Figure 1:**
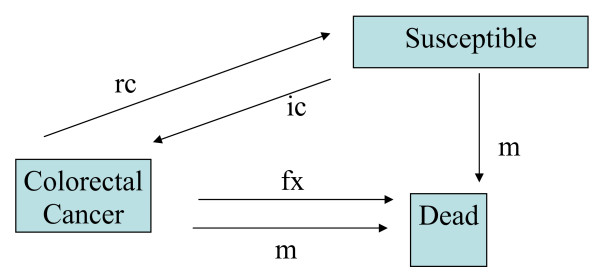
**POPMOD model of Colorectal Cancer**. ic is colorectal cancer incidence rate, rc is colorectal cancer remission rate, m is background mortality rate, fx is colorectal cancer mortality rate.

Based on the estimates obtained from the epidemiological model, population-level intervention effectiveness was estimated using a population state transition model [78] simulating the regional population demography (Additional file 1) and the effects of the disease in question (Fig. 1).

Health state valuations (HSV), based on data used by the WHO to estimate the Global Burden of Disease (GBD) (Personal Communication K. Shibuya, WHO), were specified (on a 0-1 scale, where 1 equals full health) for time spent in susceptible or diseases states (0.8 for diagnosis and treatment, 0.8 for watchful waiting whether in a treated or not treated person, 0.25 for metastasis and 0.19 for terminal stage). In keeping with the GBD methodology, no additional disability weight was ascribed to a case after a person had survived five years unless they possessed a permanent colostomy, which was ascribed a HSV of 0.79 as a result of perforation of the colon occurring in 0.129% [48,56-59,64,66,70,71] of colonoscopies and an assumed 9% of all colorectal cancer related surgical procedures.

Based on the categories "treated and survived", "treated and died", "not treated and died", "died from background causes", the weighted average age-and-sex-specific health state valuation were calculated for the null scenario, the complete treatment scenario and the scenarios of screening with treatment.

For each scenario, the initial population data inputted into the model, was projected forward for a period of 100 years. The difference in the total number of healthy years between each intervention simulation and the baseline (null) scenario was the estimate of population-level health gain due to the intervention. In keeping with the standardized WHO-CHOICE methodology DALYs averted were calculated and are discounted at a rate of 3% per annum and are age-weighted by weighting a year of healthy life lived at younger and older ages lower than a year lived at other ages [81].

### Costs of Colorectal cancer interventions

Costs for the 10-year intervention implementation period were discounted at 3% and expressed in international dollars ($I) at year 2000 price levels. An international dollar is a unit of currency with purchasing power equivalent to a US dollar in the USA [11]. Costs in local currency units were converted to international dollars using purchasing-power-parity (PPP) exchange rates. Expressing costs in international dollars facilitates more meaningful comparisons across subregions by adjusting for differences in local relative prices.

For the annual FOBT, program costs (excluding the actual costs of the FOBT), were based on an estimate of around 27 administrative posts (for notification, sending out test kits, results etc.) per 5 million population in each region in addition to a budget for media, office space and other items. Program costs for the other screening interventions and regions were adjusted to reflect the type of intervention (eg: no test kits need to be sent for sigmoidoscopy or colonoscopy), the intervention's relative frequency and the size of the target population. In less developed sub-regions (ie: regions characterized by mortality stratum D or E in reference 15) it was assumed that in the absence of a postal system, health workers would deliver the FOBT kits by hand and the kits would be returned to laboratories en bloc from the district health centers. In addition, each program had a provision for staff training and national posts for management, monitoring and evaluation based on the British NHS Cancer Screening Programs.

Quantities (manpower time, rooms, drugs, disposable and reusable equipment) for screening tests and treatment procedures were based on the WHO Collaborating Centre for Essential Health Technologies data base. Provision was made for pre-operative work-up tests such as CT scan and Chest X-rays [82]. If further data was available from published literature we adjusted the manpower time to be in accord with the published literature. For example, recent literature estimated 145.5 and 165.5 minutes average time for a colectomy [83] with and without colostomy respectively, bringing the cost of the operation up to $I845 and $I906 in AmrA, including a provision for an assumed 10% of procedures to be carried out under combined spinal-epidural anaesthesia [84]. Proctectomies were assumed to take 60 minutes longer than colectomies.

Estimates of direct cost per test (excluding programme and training overheads) for the AmrA region of $I 4, $I 71 and $I 190 for FOBT, diagnostic sigmoidoscopy and diagnostic colonoscopy, respectively, were similar to those reported in Holland [65] and Israel [72]. Colonoscopy costs included not only preparation, obtaining consent, procedure and recovery time but also one full hour for pre-screening counseling. Discounted costs of lifetime care for perforated colon were assumed to be around $I 13,000 [58], consisting of hospitalization, anesthesia, colon suture, electrocardiography, X-ray and initial care costs.

Unit costs of secondary and tertiary hospital in-patient days and out-patient visits were based on an econometric analysis of a multinational dataset of hospital costs [3]. Prices of pharmaceuticals were obtained from international [85] or from British National Health Service prices [86] adjusted to year 2000 price levels. Annual resource use per case on a stage-specific basis (i.e. initial, watchful waiting and terminal) was based on Medicare data from the USA (personal communication, Martin L. Brown, Health Services and Economic Branch, National Cancer Institute, Bethesda MD.). Liver function tests were assumed to be given monthly for one year, CT scans annually for three years, carcino-embrionic antigen tests every 6 months for three years, chest X-rays annually for 3 years and follow-up colonoscopies biannually [49].

Average unit costs (see Additional file 2) were multiplied by the number of units of care required by the sub-regional population, to estimate the total annual intervention cost.

### Decision rules

An intervention was termed very cost-effective and cost-effective if the cost per DALY was less than the per capita GNP or between 1 and 3 times per capita GNP, respectively. If the cost per DALY was more than three times the GNP per capita, then the intervention was regarded as not cost effective [87]. Sensitivity analyses were performed to generate costs per DALY under scenarios with no age-weighting and without discounting at 3% per annum.

For each region, graphical plots for each intervention of DALYs gained against costs were made in order to identify the most cost-effective interventions. The lines joining the loci of the most cost-effective points form the "expansion path", which reveals the mix of interventions that would be chosen on cost-effective grounds for any given level of resource availability [5].

## Results

We present the results for three representative regions (Table 2]: AmrA, characterised according to the WHO rubrick [1] by high income ($I 31,477 GNP per head) and low child and adult mortality, EurC, characterised by low income ($I 6,916 GNP per head), low child and high adult mortality and AfrE, characterised **by very low **income ($I 1,576 GNP per head), high child and very high adult mortality.

**Table 2:** Average Cost per DALY in relation to the null of interventions to reduce Colorectal Cancer in selected WHO subregions

	AFRE			AMRA			EURC		
	
Intervention	COST	DALYS saved	COST per DALY	COST	DALYS saved	COST per DALY	COST	DALYS saved	COST per DALY
	I$ (mill)		I$	I$ (mill)		I$	I$ (mill)		I$
Current Scenario (a)	116	27,546	4,206	64,937	14,135,241	4,594	4,677	1,801,461	**2,596**

FOB1	4,193	98,525	42,557	11,745	1,603,126	7,326	5,440	548,649	9,915

FOB2	2,222	66,502	33,410	6,448	1,082,872	5,954	2,954	370,055	7,984

SIG5	1,407	90,077	15,620	6,807	1,463,474	4,651	2,716	492,378	5,516

COL10	1,561	117,977	13,231	7,858	2,020,645	3,889	3,070	661,542	4,641

FOB1SIG5	4,915	121,374	40,491	15,989	1,969,383	8,119	7,069	665,773	10,617

FOB50	380	12,222	31,055	1,082	198,064	5,465	526	59,843	8,786

SIG50	531	41,910	12,669	2,446	679,950	3,597	984	205,514	4,786

COL50	1,040	91,092	11,415	5,027	1,491,646	**3,370**	1,980	449,523	4,404

FOBSIG50	478	46,981	10,183	3,032	762,325	3,977	860	230,391	3,734

RX	1,394	837,066	**1,666**	73,225	14,991,673	4,884	12,145	4,200,308	**2,891**

FOB1RX	5,461	912,458	5,984	77,579	16,300,533	4,759	16,481	4,630,614	3,559

FOB2RX	3,524	890,163	3,959	74,346	15,929,042	4,667	14,328	4,507,099	3,179

SIG5RX	2,706	896,387	3,019	75,839	15,864,896	4,780	14,100	4,518,157	3,121

COL10RX	2,844	909,822	**3,126**	76,031	16,131,444	4,713	14,301	4,604,861	**3,106**

FOB1SIG5RX	5,110	922,577	**5,539**	74,917	16,382,245	**4,573**	15,584	4,672,483	3,335

FOB50RX	1,758	850,239	2,067	74,130	15,200,680	4,877	12,633	4,275,966	2,954

SIG50RX	1,897	867,915	2,185	74,793	15,433,538	4,846	12,975	4,357,438	**2,978**

COL50RX	2,377	899,415	**2,643**	76,236	15,881,346	4,800	13,780	4,509,360	**3,056**

FOBSIG50RX	2,188	871,888	2,509	75,660	15,494,325	4,883	14,712	4,377,287	**3,361**

FVCAMP	275	7,618	36,074	366	84,085	4,354	360	17,804	20,222

FVCAMPRX	1,681	842,102	1,996	73,476	15,037,102	4,886	12,513	4,210,885	2,972

DRE1	818	9,438	86,676	2,370	153,861	15,401	1,008	52,939	19,038

DRE1RX	2,421	846,382	2,861	75,207	15,145,147	4,966	13,299	4,259,810	3,122

Cost-effective threshold			4,728			94,431			20,748
Very cost-effective threshold			1,576			31,477			6,916

(Discounted at 3% per annum & Age-Weighted).

Note: Interventions that fall on expansion path are in bold type.

(a) The current scenario represents the interventions which are currently being provided in the sub-regions. This differs from the reference strategy, the null, where no intervention or treatment is provided.

**Figure 2:**
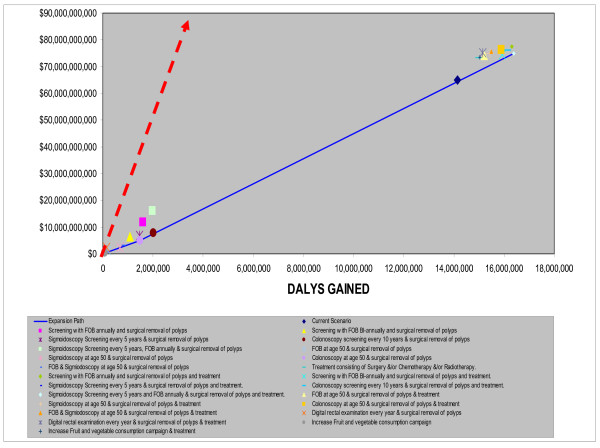
**Cost-Effectiveness of Interventions for colorectal Cancer in AMRA sub-region**. Note: Interventions falling above the broken line are not cost-effective, Interventions falling between the broken and continuous line are cost-effective. Interventions falling below the continuous line are very cost effective.

**AmrA (**Canada, United States Of America, Cuba)Two main groups of interventions emerge in the AmrA region (Fig. 2) which are based on the results presented in Table 2.

The first consists of the screening interventions (with surgical removal of polyps) in an environment where treatment (in the form of surgery, radiotherapy and chemotherapy) was not provided. Campaigns to increase fruit and vegetable consumption are close to the expansion path (indicating the lowest costs per DALY for that level of resource usage) despite the omission of benefits from decreases in diseases besides colorectal cancer. However such an intervention only accounted for a small absolute reduction in DALYs. One-off colonoscopy at age 50 falls on the expansion path. However, because of the variability inherent in both the effectiveness (i.e: increase in DALYS saved) and cost estimates, it is unlikely that there are any significant differences in the cost per DALY generated by any of the screening methods, implying no one single method can be thought of as dominant. Interventions in this group are all very cost effective (including the use of the DRE) shown by their falling to the right of the broken-arrow line indicating the points where the cost per DALY are exactly equal to the GDP per capita.

**Figure 3:**
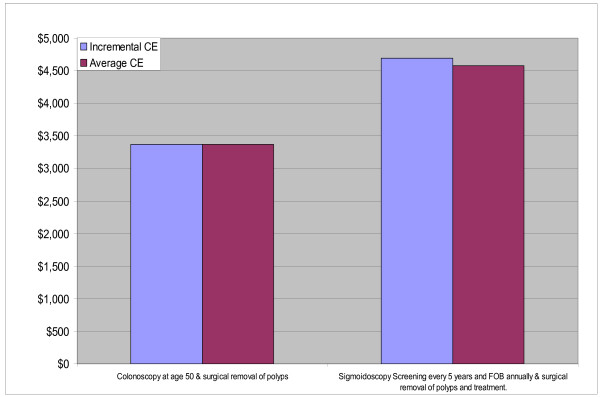
**Interventions falling on Expansion path for AMRA sub-region**.

The second group consists of screening interventions with treatment. Interventions in this group cost more and yield more DALYs than interventions in the first (no-treatment) group, although they are still very cost effective. In this treatment scenario, annual FOBT combined with sigmoidoscopy every five years is now indicated by being on the expansion path (Fig. 3), having an incremental cost effectiveness ratio (ICER) well below the GNP per head threshold. Once again due to variations in the estimates, no single intervention combined with treatment can be thought of as being superior to the others.

**EurC (**Belarus, Estonia, Hungary, Kazakhstan, Latvia, Lithuania, Republic of Moldova, Russian Federation, Ukraine)

**Figure 4:**
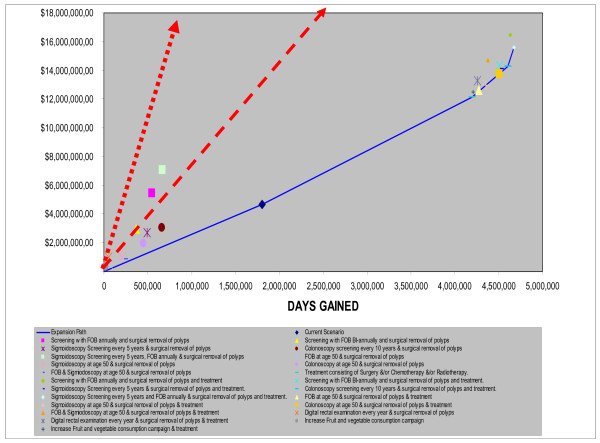
**Cost-Effectiveness of Interventions for colorectal Cancer in EURC sub-region**. Note: Interventions falling above the broken line are not cost-effective, Interventions falling between the broken and continuous line are cost-effective. Interventions falling below the continuous line are very cost effective.

Again two main distinct groupings emerge (Fig. 4) which are based on the results presented in Table 2. However the no-treatment group is less homogeneous than in AmrA. The one off screening interventions at age 50 (colonoscopy, sigmoidoscopy with and without FOBT) were very cost-effective as was sigmoidoscopy every five years and colonoscopy every ten years. The other screening interventions (including the DRE) were just cost-effective, falling between the dotted and dashed lines representing the three and one times the GNP per head thresholds respectively.

**Figure 5:**
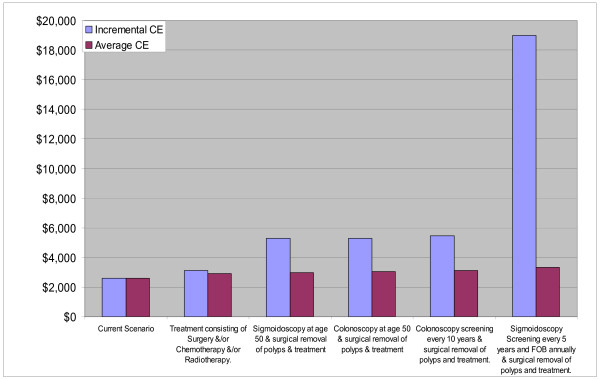
**Interventions falling on Expansion path for EURC sub-region**.

All the screening interventions with treatment are very cost effective falling to the right of the dashed line. As more resources become available the expansion path shifts interventions in the current scenario (characterised by medium levels of treatment coverage) to universal treatment, then to sigmoidoscopy at age 50, colonoscopy at age 50, to colonoscopy screening every 10 years, gaining the most DALYS when a combined FOBT and sigmoidoscopy programme is complemented by full treatment (Fig. 5).

The ICER of moving along the expansion path showed that all the interventions up to supplying colonoscopies every 10 years to be very cost-effective. However expansion to a combined FOBT and sigmoidoscopy intervention might be considered as just cost effective as its ICER is between one and three times the per capita GNP. Once again, no single intervention combined with treatment dominates.

**AfrE (**Botswana, Burundi, Central African Republic, Congo, Côte d'Ivoire, Democratic Republic Of The Congo, Eritrea, Ethiopia, Kenya, Lesotho, Malawi, Mozambique, Namibia, Rwanda, South Africa, Swaziland, Uganda, United Republic of Tanzania, Zambia, Zimbabwe)

**Figure 6:**
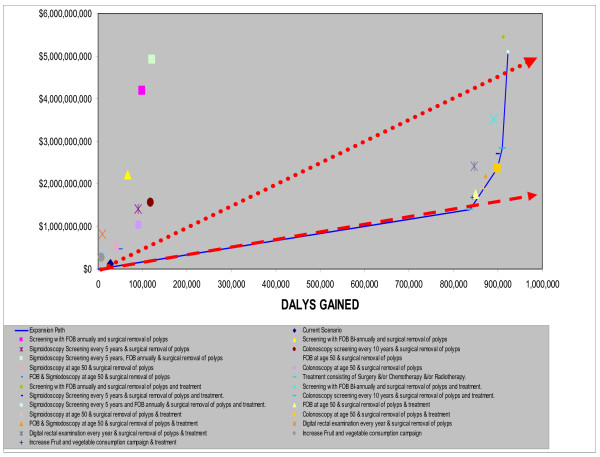
**Cost-Effectiveness of Interventions for colorectal Cancer in AFRE sub-region**. Note: Interventions falling above the broken line are not cost-effective, Interventions falling between the broken and continuous line are cost-effective. Interventions falling below the continuous line are very cost effective.

As a result of cost differentials associated with programme implementation, there is a wide range of costs between screening programmes without treatment (Fig. 6) which are based on the results presented in Table 2. All of the screening interventions (in the no treatment scenario) were found to be not cost effective (ie: they fall to the left of the dotted arrowed line) due primarily to the lower incidence of the disease in the region.

**Figure 7:**
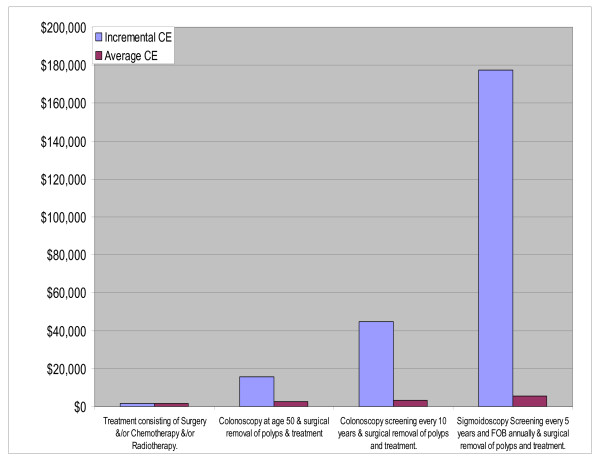
**Interventions falling on Expansion path for AFRE sub-region**.

Universal treatment, (ie: 100% treatment scenario), colonoscopy at age 50 (with polyp removal), colonoscopy every 10 years and sigmoidoscopy every five years combined with annual FOBT with treatment appear on the expansion path, only the first three being cost-effective (ie: falling between the dotted and dashed lines) (Fig. 7). However, using the yardstick that any intervention whose ICER is in excess of three times the per capita GNP is not cost effective, then adding any of the screening programmes to treatment will not be considered as being cost effective. Screening persons aged under 50 years old yielded less favourable cost-effectiveness ratios than commencing screening at age 50 years.

### Sensitivity analysis

Applying age weights to health effects is not without controversy [79]. Removing age weighting results in an overall decrease in the cost per DALY of interventions (Additional file 3). In AmrA, Colonoscopy every 10 years (with polyp removal) joins the expansion path, in EurC annual FOB with sigmoidoscopy every 5 years (with polyp removal and treatment) joins the expansion path. For AfrE, sigmoidoscopy at age 50 (with polyp removal and treatment) joins the expansion path. The removal of age-weighting means that treating everybody becomes very cost-effective, falling below the GNP per capita threshold.

When both discounting and age-weighting are removed (Additional file 4), costs per DALY fall still further. The expansion path of AmrA consists of colonoscopy at age 50, colonoscopy every 10 years, and sigmoidoscopy every five years combined with annual FOBT (with polyp removal and treatment). In EurC the expansion path consists of treatment only, colonoscopy every 10 years with treatment and sigmoidoscopy every five years combined with annual FOBT with treatment. In AFRE, sigmoidoscopy at age 50 with and without annual FOBT joins universal treatment as being very cost-effective.

## Discussion

Cost-effectiveness estimates for developed countries (mainly USA and countries in Europe) have reported a wide range of incremental costs per life year for the colorectal cancer interventions examined in this, over and above that of treatment alone. Baseline costs per life year (in USD at 2000 price levels) relative to a no screening strategy for both the one-off and repetitive screening vary considerably. Seven studies reported costs per life year of $20,000 or more [49,58-60,69-71], eight reported in the range $10,000 - $19,999 [47,48,56-59,62,70], three between $5,000 - $9,999 [55,57,58] and three between $0-$4999 [56,64,88]. Some models even indicated incremental cost-savings (alongside effectiveness gains), both for one-off [72] and repeated screening interventions [56,72].

Such wide ranges in the cost-utility ratio and the subsequent lack of dominance of any one screening mode (reflected by different rankings between the screening methods) are a consequence of the heterogeneous nature of the model specifications (on the effectiveness side), variations in the duration of operation of screening programmes and wide variations in the estimates of the interventions unit costs. These ranged considerably, with sigmoidoscopy costing between six [69] to 27 times [70] that of FOBT, colonoscopy costing between 10 [72] to 100 times that of FOBT [57,66]. Our estimates showed sigmoidoscopy and colonoscopy costs to be between 18 and 48 times that of the basic FOBT test. Additional research is clearly needed on estimating screening programme costs as these considerably affect the cost per DALY outcomes, especially in developing countries.

Our epidemiological model was simpler than most of published models and lacked refinements such as taking into account the distribution of multiple polyps and biphasic polyp dwelling times [68]. In addition, our model did not include provision for more frequent screening of persons identified as having a polyp or with a familial history of CRC.

While our model was based on regional specific age and gender distributions, information on age-specific polyp incidence distribution was based only on available USA data. If a developing country's relative distribution of polyp incidence is skewed to a younger age than the USA, then this would mean that the screening programme would have a lower efficacy (unless screening ages were adjusted) and so our cost-effectiveness ratios are likely to be upwardly biased. On the other hand, our somewhat optimistic compliancy assumption that all persons screened positive on FOBT or Sigmoidoscopy would receive a subsequent colonoscopy served to downwardly bias the presented cost per DAYS, as some cases are likely to be lost to follow-up in practice.

Our estimated cost-effectiveness ratios for the incremental addition of various screening programmes to treatment (for comparability with the literature, discounted at 3% but not age-weighted; see Additional file 2) in AmrA of $800-$2,200 per DALY (for screening from age 50 to 80) and $2,400-$3,500 (for one-off interventions) fell towards the lower end of the wide spectrum of results presented in the opening paragraph of this discussion. This could be attributed to a variety of causes, such as the use of economic costs as opposed to charges or prices for procedures and the longer duration (relative to much of the literature) of the time-horizon used to evaluate our interventions. Inclusion of morbidity gains in our denominator reduced still further our ratios, although this was somewhat counterbalanced by our use of healthy life years in the denominator (as opposed to life years as reported in most of the literature).

In general the following policy recommendations could be inferred from our results. In subregions characterised by high income, low mortality and high existing treatment coverage (eg: AmrA subregion), both the incremental (ie: the additional cost per DALY of adding a screening programme to the existing treatment provision) and general (ie: compared to the null) cost-effectiveness results point to the very cost-effective nature of the screening intervention, although no particular specific intervention is indicated as being dominant.

In subregions characterised by low income, low mortality with existing treatment coverage around 50% (eg: EurC subregion), expanding treatment with or without combining it with screening programmes appears to be cost-effective or very cost-effective. Abandoning treatment in favour of funding screening programmes that would operate in a no treatment scenario would not be a cost-effective measure.

In subregions which have low income, low mortality and low treatment levels (eg; AfrE subregion), the best strategy is to provide resources to treat persons with colorectal cancer as opposed to providing a screening programme.

Use of the DRE alone was very cost-effective in the AmrA subregion and cost-effective in EurC. However it has low efficacy and provides a low level of absolute DALY gains. Despite its low technological approach, DRE is not cost-effective in the low incidence AfrE subregion. However, the low cost simple DRE (both with and without follow-up diagnostic colonoscopy), suffers from a lack of direct evidence that the exam reduces mortality from colorectal cancer, our modelled efficacy being based on a single no-significant result (OR = 0.96, 95% CI 0.56-1.70) from a case control study from northern California [18]. Sensitivity is low, as fewer than 10% of colorectal cancer causing polyps are within reach of the examining finger. There is therefore no solid basis for recommending this method as a stand alone screening intervention for colorectal cancer. Moreover, a comprehensive assessment of this intervention should include its effect on reducing mortality from prostate cancer.

Like the digital rectal examination, the introduction of a fruit and vegetable campaign gains fewer DALYS then any of the screening interventions. Such a campaign was however found to be very cost effective in AmrA even without the inclusion of additional benefits resulting from decreases in other diseases. The fruit and vegetable campaign was marginally cost-effective in EurC, but was not cost-effective at all in AfrE. However, the external validity of applying the effectiveness results from one health promotion campaign in Australia [55] to other regions of the world can be questioned.

The 20% price subsidy will actually be less effective than health promotion in increasing fruit and vegetable consumption, and hence in reducing colorectal cancer. Estimating the economic cost of such an intervention is beyond the scope of this paper. Moreover, an evaluation of such an intervention requires the effectiveness benefits of reductions in heart disease, stroke and other cancers to be included. The cost per DALY, however, might be underestimated since the income freed up by the food subsidy might result in increased consumption of red meat, which could well be a risk factor for colorectal cancer [26,27].

Our use of a single measure of relative risk reduction for each age and gender specific intervention simplifies the reality, where the risk reduction and subsequent cost-effectiveness ratio can differ depending on the socio-economic context of the screening subgroups [88]. One could also argue that present models on colorectal cancer screening are inadequate for aiding public health decision-making because the efficacy evidence is too preliminary for any screening modality other than FOBT [89]. Lack of such randomized controlled trial evidence precluded an evaluation of immunological FOBT testing, which is likely to yield lower costs per QALY than guaiac-based tests [61], due to the higher sensitivity of immunological FOBT to detect colorectal neoplasms [90-92] outweighing increased unit costs. Our analysis also did not consider the options of targeting screening to high-risk populations which despite reducing the overall DALY gains may well increase the cost-effectiveness of the interventions [51].

The CEAs presented represent first order point estimates of various interventions. When allowance is made for variations of the estimates by sensitivity analysis then it appears there is no clear discernable difference in terms of cost-effectiveness between the major screening options. Perhaps another key factor in deciding what option or mix of options to adopt, would be those interventions which have the highest expected attainable coverage rates. Adoption of screening policies between the ages of 50 and 80 will only eradicate a small portion (between 14%-24%) of the existing colorectal cancer burden, since the application of the compliancy rate to the intervention efficacy, even in the case of colonoscopy every 10 years will only result in a 24.1% reduction in incidence. One-off screening policies will reduce an even lower percentage of the total disease burden.

The cost-effectiveness ratios are insensitive to changes in compliancy, particularly in scenarios that include access to treatment and where programme operational overheads are low. Basically, the increases or decreases in programme efficacy resulting from changes in compliancy are somewhat counterbalanced (except for the programme cost overheads) by decreases or increases in costs.

The cost-effectiveness ratios are also biased upwards (or downwards) to the extent that transport costs and costs of work lost due to treatment exceed (or are exceeded by) the transport costs and costs of work lost due to screening [93,94].

Decisions to adopt interventions are usually made by health services and governments at the country level. Although the model used in this analysis is based on regions, the parameters defining the model can be adjusted to country level.

## Abbreviations

CEA: Cost Effectiveness Analysis; CFR: Case Fatality Rate; CHOICE: Choosing Interventions that are Cost-Effective; DALY: Disability Adjusted Life Year; DRE: Digital Rectal Exams; EIP: Evidence and Information in policy; FOBT: Fecal Occult Blood Test; GBD: Global Burden of Disease; GCEA: Generalized Cost Effectiveness Analysis; GNP: Gross National Product; HSV: Health Status Valuation; ICER: Incremental Cost Effectiveness Ratio; Ln: Natural Logarithm; POPMOD: A state transitional population model; PPP: Purchasing Power Parity; HO: World Health Organization.

## Competing interests

This study was undertaken by persons who were on the salaried staff of the WHO at the time of the study. None of the authors have any competing interests.

## Authors' contributions

GG collected data, designed, calculated and wrote up the study. SL provided technical assistance with the modelling and commented on earlier drafts. JL provided technical assistance with the modelling and wrote up the study. BJ provided assistance on the costings. CS provided assistance related to the medical and epidemiological aspects.

All authors have read and approved the final manuscript.

## Supplementary Material

Additional file 1 Selected Variables by Region. Click here for file

Additional file 2 Unit Cost ($ International) by Selected Regions. Click here for file

Additional file 3 Average Cost per DALY in relation to the null of interventions to reduce Colorectal Cancer in selected WHO sub-regions. Click here for file

Additional file 4 Average Cost per DALY in relation to the null of interventions to reduce Colorectal Cancer in selected WHO sub-regions. Click here for file
