# Supplementary material for: Prevention, screening and treatment of colorectal cancer: a global and regional generalized cost effectiveness analysis
Source: Cost Eff Resour Alloc. 2010 Mar 17;8:2. doi: 10.1186/1478-7547-8-2 (PMC2850877; doi:10.1186/1478-7547-8-2)
Supplement: Additional file 1 — Selected Variables by Region. [file 1478-7547-8-2-S1.DOC]

| **Additional file 1: Selected Variables by Region** | | | | | | | | |  | | |  | |  |  |
| --- | --- | --- | --- | --- | --- | --- | --- | --- | --- | --- | --- | --- | --- | --- | --- |
|  |  | |  |  | | |  | |  | | |  | |  |  |
|  |  | |  | **AFRE** | | |  | | **AMRA** | | |  | | **EURC** |  |
|  |  | |  |  | | |  | |  | | |  | |  |  |
| Popn. Millions (2000) (a) | | Total | | 402.0 | | |  | | 343.4 | | |  | | 236.9 |  |
| % Aged 50-79 (a) | |  | | 9.4% | | |  | | 26.2% | | |  | | 28.6% |  |
| Treatment coverage at age 65 (b) | | | | 6.7% | | |  | | 95.0% | | |  | | 50.0% |  |
|  |  | |  |  | | |  | |  | | |  | |  |  |
|  |  | |  | males | | | females | | males | | | females | | males | females |
| Background Mortality/1000 | | | |  | | |  | |  | | |  | |  |  |
| at age 65 (a) |  | |  | 46.6 | | | 32.2 | | 16.7 | | | 10.8 | | 50.4 | 19.1 |
|  |  | |  |  | | |  | |  | | |  | |  |  |
| **Colorectal Cancer** | |  | |  | | |  | |  | | |  | |  |  |
|  |  | |  |  | | |  | |  | | |  | |  |  |
| Incidence/1000 (b) | |  | |  | | |  | |  | | |  | |  |  |
| 45-54 yrs |  | |  | 0.20 | | | 0.13 | | 1.02 | | | 0.81 | | 0.53 | 0.50 |
| 55-59 yrs |  | |  | 0.52 | | | 0.33 | | 2.58 | | | 1.79 | | 1.47 | 1.06 |
| 60-64 yrs |  | |  | 0.68 | | | 0.43 | | 3.36 | | | 2.28 | | 1.94 | 1.34 |
| 65-70 yrs |  | |  | 1.14 | | | 0.43 | | 4.26 | | | 2.28 | | 2.43 | 1.34 |
| 70-79 yrs |  | |  | 1.59 | | | 0.83 | | 5.17 | | | 3.93 | | 2.93 | 2.07 |
| 80+ yrs |  | |  | 3.36 | | | 1.61 | | 6.18 | | | 2.54 | | 2.94 | 1.79 |
|  |  | |  |  | | |  | |  | | |  | |  |  |
| Prevalence/1000 (b) | |  | |  | | |  | |  | | |  | |  |  |
| 45-54 yrs |  | |  | 0.89 | | | 0.44 | | 6.99 | | | 5.69 | | 3.39 | 3.47 |
| 55-59 yrs |  | |  | 2.22 | | | 1.06 | | 17.40 | | | 12.31 | | 8.63 | 6.77 |
| 60-64 yrs |  | |  | 2.88 | | | 1.37 | | 22.60 | | | 15.62 | | 11.24 | 8.42 |
| 65-70 yrs |  | |  | 4.02 | | | 1.73 | | 27.75 | | | 20.68 | | 11.99 | 9.32 |
| 70-79 yrs |  | |  | 5.16 | | | 2.08 | | 32.91 | | | 25.74 | | 12.74 | 10.22 |
| 80+ yrs |  | |  | 9.48 | | | 3.48 | | 36.89 | | | 15.74 | | 8.83 | 6.44 |
|  |  | |  |  | | |  | |  | | |  | |  |  |
|  | | | | | |  | |  | |  | | |  | | |
| (a) Source: WHO demographic data (1). | | | | | |  | |  | |  | | |  | | |
| (b) Source: WHO Global Burden of Disease Data. | | | | | | | | | |  | | |  | | |
|  | | | | | | | | | | |  | | | | |
|  | | | | |  | | |  | |  | | | | | |
