# Supplementary material for: Prevention, screening and treatment of colorectal cancer: a global and regional generalized cost effectiveness analysis
Source: Cost Eff Resour Alloc. 2010 Mar 17;8:2. doi: 10.1186/1478-7547-8-2 (PMC2850877; doi:10.1186/1478-7547-8-2)
Supplement: Additional file 2 — Unit Cost ($ International) by Selected Regions. [file 1478-7547-8-2-S2.DOC]

| **Additional file 2: Unit Cost ($ International) by Selected Regions** | | | | | |  |
| --- | --- | --- | --- | --- | --- | --- |
|  |  |  | |  |  |  |
|  |  |  | | **AFRE** | **AMRA** | **EURC** |
|  |  |  | |  |  |  |
| Digital Rectal Examination | | | 1.47 | | 5.26 | 2.03 |
| FOBT |  |  | | 1.97 | 4.83 | 2.39 |
| Sigmoidoscopy, flexible diagnostic | | | 33 | | 76 | 38 |
| Colonoscopy, flexible diagnostic | | | 96 | | 208 | 105 |
| Colonoscopy with lesion removal | | | 103 | | 249 | 116 |
|  |  |  | |  |  |  |
| Radiotherapy session | | | 50 | | 122 | 57 |
| Chemotherapy, session | | | 124 | | 122 | 114 |
|  |  |  | |  |  |  |
| Partial colectomy with anastomosis | | | 137 | | 513 | 187 |
| Partial colectomy with colostomy | | | 155 | | 582 | 211 |
| Total colectomy with ileostomy | | | 153 | | 581 | 210 |
|  |  |  | |  |  |  |
| Partial protectomy with reservoir | | | 206 | | 790 | 284 |
| Partial protectomy with anastomosis | | | 189 | | 781 | 259 |
| Complete protectomy with colostomy | | | 205 | | 789 | 282 |
