# Supplementary material for: Prevention, screening and treatment of colorectal cancer: a global and regional generalized cost effectiveness analysis
Source: Cost Eff Resour Alloc. 2010 Mar 17;8:2. doi: 10.1186/1478-7547-8-2 (PMC2850877; doi:10.1186/1478-7547-8-2)
Supplement: Additional file 3 — Average Cost per DALY in relation to the null of interventions to reduce Colorectal Cancer in selected WHO sub-regions. [file 1478-7547-8-2-S3.DOC]

| **Additional file 3: Average Cost per DALY in relation to the null of interventions to reduce Colorectal Cancer in selected WHO sub-regions.**  **(Discounted at 3% per annum & not Age-Weighted).** | | | | | | | | | |
| --- | --- | --- | --- | --- | --- | --- | --- | --- | --- |
|  |  |  |  |  |  |  |  |  |  |
|  |  | **AFRE** |  |  | **AMRA** |  |  | **EURC** |  |
| **Intervention** | COST | DALYS | COST | COST | DALYS | COST | COST | DALYS | COST |
|  |  | saved | per |  | saved | per |  | saved | per |
|  |  |  | DALY |  |  | DALY |  |  | DALY |
|  | I$ (mill) |  | I$ | I$ (mill) |  | I$ | I$ (mill) |  | I$ |
| Current Scenario | 106 | 28,779 | 3,677 | 64,937 | 19,700,564 | 3,296 | 4,672 | 2,279,524 | **2,050** |
| FOB1 | 2,196 | 143,999 | 15,252 | 11,745 | 2,469,894 | 4,755 | 5,750 | 830,327 | 6,925 |
| FOB2 | 1,210 | 97,274 | 12,439 | 6,448 | 1,669,894 | 3,861 | 3,111 | 560,326 | 5,553 |
| SIG5 | 1,187 | 129,903 | 9,139 | 6,807 | 2,226,272 | 3,057 | 2,748 | 735,782 | 3,735 |
| COL10 | 1,422 | 170,359 | 8,347 | 7,858 | 3,038,463 | **2,586** | 3,091 | 981,565 | 3,149 |
| FOB1SIG5 | 2,918 | 174,644 | 16,708 | 15,989 | 2,987,782 | 5,351 | 7,378 | 993,221 | 7,429 |
| FOB50 | 257 | 15,602 | 16,493 | 1,082 | 266,277 | 4,065 | 522 | 78,532 | 6,651 |
| SIG50 | 492 | 53,467 | 9,211 | 2,446 | 913,291 | 2,678 | 983 | 269,442 | 3,649 |
| COL50 | 1,010 | 116,516 | 8,664 | 5,027 | 2,004,412 | **2,508** | 1,979 | 590,104 | 3,353 |
| FOBSIG50 | 356 | 59,935 | 5,943 | 3,032 | 1,023,884 | 2,961 | 857 | 302,051 | 2,837 |
| RX | 1,393 | 960,794 | **1,450** | 73,225 | 21,207,448 | 3,453 | 12,145 | 5,682,099 | **2,137** |
| FOB1RX | 3,463 | 1,072,727 | 3,228 | 77,579 | 23,264,482 | 3,335 | 16,791 | 6,342,875 | 2,647 |
| FOB2RX | 2,511 | 1,039,340 | 2,416 | 74,346 | 22,675,818 | 3,279 | 14,485 | 6,151,175 | 2,355 |
| SIG5RX | 2,485 | 1,048,552 | 2,370 | 75,839 | 22,572,481 | 3,360 | 14,133 | 6,168,581 | 2,291 |
| COL10RX | 2,704 | 1,070,410 | **2,527** | 76,031 | 22,983,974 | 3,308 | 14,321 | 6,303,734 | **2,272** |
| FOB1SIG5RX | 3,112 | 1,086,870 | **2,864** | 74,917 | 23,381,619 | **3,204** | 15,894 | 6,403,078 | **2,482** |
| FOB50RX | 1,634 | 978,252 | 1,671 | 74,130 | 21,499,118 | 3,448 | 12,629 | 5,785,778 | 2,183 |
| SIG50RX | 1,858 | 1,001,746 | **1,854** | 74,793 | 21,822,646 | 3,427 | 12,974 | 5,896,880 | **2,200** |
| COL50RX | 2,342 | 1,044,027 | **2,243** | 76,236 | 22,448,942 | 3,396 | 13,779 | 6,105,302 | 2,257 |
| FOBSIG50RX | 2,027 | 1,007,037 | 2,013 | 75,660 | 21,907,998 | 3,454 | 14,707 | 5,924,027 | 2,483 |
| FVCAMP | 275 | 8,914 | 30,829 | 366 | 120,992 | 3,026 | 360 | 24,862 | 14,481 |
| FVCAMPRX | 1,681 | 966,750 | 1,739 | 73,476 | 21,274,746 | 3,454 | 12,513 | 5,697,224 | 2,196 |
| DRE1 | 381 | 13,931 | 27,319 | 2,370 | 239,336 | 9,901 | 1,069 | 80,792 | 13,228 |
| DRE1RX | 1,786 | 974,289 | 1,833 | 75,207 | 21,441,997 | 3,507 | 13,410 | 5,770,799 | 2,324 |
|  |  |  |  |  |  |  |  |  |  |
| Cost-effective threshold |  |  | 4,728 |  |  | 94,431 |  |  | 20,748 |
| Very cost-effective threshold |  |  | 1,576 |  |  | 31,477 |  |  | 6,916 |

**Note: Interventions that fall on expansion path are in bold type.**
