# Supplementary material for: Prevention, screening and treatment of colorectal cancer: a global and regional generalized cost effectiveness analysis
Source: Cost Eff Resour Alloc. 2010 Mar 17;8:2. doi: 10.1186/1478-7547-8-2 (PMC2850877; doi:10.1186/1478-7547-8-2)
Supplement: Additional file 4 — Average Cost per DALY in relation to the null of interventions to reduce Colorectal Cancer in selected WHO sub-regions. [file 1478-7547-8-2-S4.DOC]

| **Additional file 4: Average Cost per DALY in relation to the null of interventions to reduce Colorectal Cancer in selected WHO sub-regions.**  **(Undiscounted & not Age-Weighted).** | | | | | | | | | |
| --- | --- | --- | --- | --- | --- | --- | --- | --- | --- |
|  |  |  |  |  |  |  |  |  |  |
|  |  | **AFRE** |  |  | **AMRA** |  |  | **EURC** |  |
| **Intervention** | COST | DALYS | COST | COST | DALYS | COST | COST | DALYS | COST |
|  |  | saved | per |  | saved | per |  | saved | per |
|  |  |  | DALY |  |  | DALY |  |  | DALY |
|  | I$ (mill) |  | I$ | I$ (mill) |  | I$ | I$ (mill) |  | I$ |
| Current Scenario | 106 | 39,858 | 2,655 | 64,937 | 29,465,027 | 2,204 | 4,672 | 3,180,763 | **1,469** |
| FOB1 | 2,196 | 222,812 | 9,857 | 11,745 | 4,088,059 | 2,873 | 5,750 | 1,307,868 | 4,396 |
| FOB2 | 1,210 | 150,447 | 8,043 | 6,448 | 2,762,034 | 2,334 | 3,111 | 882,117 | 3,527 |
| SIG5 | 1,187 | 202,028 | 5,876 | 6,807 | 3,705,880 | 1,837 | 2,748 | 1,165,931 | 2,357 |
| COL10 | 1,422 | 270,346 | 5,260 | 7,858 | 5,111,877 | **1,537** | 3,091 | 1,576,044 | 1,961 |
| FOB1SIG5 | 2,918 | 271,798 | 10,736 | 15,989 | 4,977,963 | 3,212 | 7,378 | 1,574,620 | 4,686 |
| FOB50 | 257 | 25,460 | 10,107 | 1,082 | 469,302 | 2,306 | 522 | 132,473 | 3,943 |
| SIG50 | 492 | 87,230 | 5,646 | 2,446 | 1,608,863 | 1,520 | 983 | 454,260 | 2,164 |
| COL50 | 1,010 | 190,565 | 5,297 | 5,027 | 3,533,615 | **1,423** | 1,979 | 996,126 | 1,986 |
| FOBSIG50 | 356 | 97,784 | 3,643 | 3,032 | 1,803,679 | 1,681 | 857 | 509,243 | 1,683 |
| RX | 1,393 | 1,321,119 | **1,055** | 73,225 | 31,444,944 | 2,329 | 12,145 | 7,850,032 | **1,547** |
| FOB1RX | 3,463 | 1,502,503 | 2,305 | 77,579 | 34,992,040 | 2,217 | 16,791 | 8,937,659 | 1,879 |
| FOB2RX | 2,511 | 1,447,682 | 1,734 | 74,346 | 33,964,295 | 2,189 | 14,485 | 8,616,396 | 1,681 |
| SIG5RX | 2,485 | 1,465,845 | 1,695 | 75,839 | 33,845,612 | 2,241 | 14,133 | 8,663,967 | 1,631 |
| COL10RX | 2,704 | 1,508,600 | **1,793** | 76,031 | 34,630,068 | 2,196 | 14,321 | 8,916,551 | **1,606** |
| FOB1SIG5RX | 3,112 | 1,528,416 | **2,036** | 74,917 | 35,247,841 | **2,125** | 15,894 | 9,054,658 | **1,755** |
| FOB50RX | 1,634 | 1,348,581 | 1,212 | 74,130 | 31,932,793 | 2,321 | 12,629 | 8,011,428 | 1,576 |
| SIG50RX | 1,858 | 1,390,261 | **1,336** | 74,793 | 32,556,283 | 2,297 | 12,974 | 8,216,101 | 1,579 |
| COL50RX | 2,342 | 1,464,555 | **1,599** | 76,236 | 33,735,626 | 2,260 | 13,779 | 8,592,975 | 1,604 |
| FOBSIG50RX | 2,027 | 1,399,168 | **1,449** | 75,660 | 32,709,505 | 2,313 | 14,707 | 8,262,975 | 1,780 |
| FVCAMP | 275 | 15,114 | 18,183 | 366 | 205,965 | 1,777 | 360 | 40,423 | 8,906 |
| FVCAMPRX | 1,681 | 1,332,054 | 1,262 | 73,476 | 31,567,890 | 2,328 | 12,513 | 7,876,590 | 1,589 |
| DRE1 | 381 | 21,464 | 17,731 | 2,370 | 394,071 | 6,013 | 1,069 | 126,641 | 8,439 |
| DRE1RX | 1,786 | 1,341,782 | 1,331 | 75,207 | 31,827,953 | 2,363 | 13,410 | 7,984,232 | 1,680 |
|  |  |  |  |  |  |  |  |  |  |
| Cost-effective threshold |  |  | 4,728 |  |  | 94,431 |  |  | 20,748 |
| Very cost-effective threshold |  |  | 1,576 |  |  | 31,477 |  |  | 6,916 |

**Note: Interventions that fall on expansion path are in bold type.**
